# Editorial: Regulation of proteolysis and proteome composition in plant response to environmental stress, volume II

**DOI:** 10.3389/fpls.2024.1439668

**Published:** 2024-06-13

**Authors:** Mateusz Labudda, Shaojun Dai, Zhiping Deng, Ling Li

**Affiliations:** ^1^ Department of Biochemistry and Microbiology, Institute of Biology, Warsaw University of Life Sciences-SGGW, Warsaw, Poland; ^2^ China Development Center of Plant Germplasm Resources, College of Life Sciences, Shanghai Normal University, Shanghai, China; ^3^ Institute of Virology and Biotechnology, Zhejiang Academy of Agricultural Sciences, Hangzhou, China; ^4^ Department of Biological Sciences, Mississippi State University, Starkville, MS, United States

**Keywords:** abiotic stress, biotic stress, environmental stress, protease, protein post-translational modification

Plants are capable of triggering defense responses against environmental stresses. Among these responses, the regulation of proteolysis, and the quality and composition of proteins, are of fundamental importance. Protein post-translational modifications (PPTMs) participate in responses of plants to environmental stresses to permit the regulation of stress and defense signaling. The biochemical mechanisms of PPTMs are currently a key topic of plant stress biochemists because they constitute one of the fastest and earliest molecular reactions to abiotic and biotic stressors. PPTMs enhance proteome diversity and expand the plant’s ability to respond to external stimuli. They influence gene expression, signal transduction pathways, and enzymatic activities, all while maintaining low energy costs for the plant organism.

This Research Topic aimed to widen the understanding on proteome diversity in plants using an interdisciplinary approach. The Research Topic contains five papers from fields across abiotic stress (such as heat stress, oxybenzone stress and high-light-intensity conditions) and biotic stress (such as infections with *Spongospora subterranea* and *Fusarium graminearum*).

Three articles published in this Research Topic focus on PPTMs such as phosphorylation, lysine 2-hydroxyisobutyrylation, and succinylation.

Balotf et al. used phosphoproteomics to study how susceptible (Iwa) and resistant (Gladiator) potato cultivars react to *S. subterranea* infestation. This study revealed 2,640 phosphosites corresponding to 1,498 phosphoproteins, because some proteins contained multiple phosphorylation sites. Moreover, it has been shown that the potato cultivars and the dissimilarity between infected and uninfected plants were the primary origins of polarity in phosphoproteomes of potato leaves. In the resistant Gladiator cultivar, alterations in abundance of 368 phosphosites were statistically significant, of which 229 were up-regulated, and 139 were down-regulated in infected plants. In the susceptible Iwa cultivar, 590 phosphosites were significantly altered, of which 435 were enhanced and 155 were diminished. Based on the phosphopeptide profiling, which revealed a significant increase in Gladiator plants, the main effect of *S*. *subterranea* infection at the phosphoproteome level were hormone signaling, phosphorelay signal transduction, and defense responses involving cell wall thickening and callose deposition. In contrast, in Iwa plants, infection led to a significant enhancement of transport activities, specifically inorganic anion transport and transmembrane transporter activity.

Following the work of Zhang et al., it is noticeable that lysine 2-hydroxyisobutyrylation (Khib) has a key role in protein biosynthesis, transcriptional regulation, and cellular metabolism in plants. In this research, the western blot analysis revealed that Khib level was enhanced significantly after *F. graminearum* infestation, and 2,066 Khib modified sites on 728 proteins were observed in *Zea mays* plants, including 24 Khib sites on core histones. Moreover, it was found that Khib-modified proteins were present in nucleus, cytoplasm, and chloroplasts. A subsequent proteomic study of the defense response of maize plants to *F. graminearum* infection discovered that, Khib took part in plant resistance to fungal infection by regulating several metabolic pathways. These include the Embden–Meyerhof–Parnas pathway, the Krebs cycle, protein biosynthesis, and peroxisomal and secondary metabolic processes, such as synthesis of benzoxazinoids, phenylpropanoids, jasmonic acid, tyrosine, and tryptophan in *F. graminearum*-infected *Z. mays* plants. Furthermore, Zhang et al. also showed that Khib-modified histones were engaged in regulating the expression of pathogenesis-related proteins, highlighting their role in the plant’s defense mechanisms.

Oxybenzone (OBZ) is one of the ultraviolet (UV) absorbents that has harmful influence on plants. Li et al. examined the role of lysine succinylation (Ksuc) in proteins during OBZ-induced stress in *Brassica rapa* L. ssp. *chinensis* plants. A total of 1,276 Ksuc sites on 507 proteins were noted. Among these sites, 181 Ksuc-changed proteins were hypersulfinylated/succinylated in OBZ-exposed pakchoi plants. Ksuc-modified proteins were localized in the cytoplasm, chloroplasts, and mitochondria and were associated with primary metabolic processes. These processes included reactive oxygen species (ROS) detoxification, stress defense responses, bioenergetics, biosynthesis of amino acids, histone modifications, regulation of photosynthesis, the Embden–Meyerhof–Parnas’s pathway, and the Krebs cycle.

The next two articles shift focus from PPTMs and present sucrose metabolizing enzymes in *Saccharum officinarum* plants under heat stress, and the chloroplast-located intramembrane protease (S2P2) and its impact on the stoichiometry and functioning of the photosynthetic apparatus in Arabidopsis plants.

Mehdi et al. observed several significant changes in sugarcane plants subjected to heat stress. They reported a decrease in the activity of sucrose metabolizing enzymes, sucrose content and sugar recovery rate. Concurrently, there were increased levels of proline, malondialdehyde (a marker for lipid peroxidation), hydrogen peroxide, and electrolyte leakage. Notably, this is the first report of different invertase isozyme molecular weight proteins, such as those with molecular weights of 67, 134, and 160 kDa that were induced under heat stress, suggesting that these enzymes exhibit differential activity at different stages of development.

Ciesielska et al. demonstrated that S2P2 is a thylakoid protein, playing a significant role in maintaining the proper functioning of chloroplasts, especially under high-light-intensity conditions. The lack of S2P2 in Arabidopsis chloroplasts resulted in a significant drop in the level of photosystem I and photosystem II core proteins: PsaB, PsbA, PsbD, and PsbC, and polypeptides in the main light-harvesting antenna complex II (LHC II), Lhcb1 and Lhcb2, as well as the minor, peripheral antenna system polypeptides Lhcb4 and Lhcb5. These alterations occurred jointly with a decline in the number of photosystems II–LHC II supercomplexes. The consequence of these changes was a greater sensitivity of *s2p2* mutant plants to photoinhibition.

Taken together, this Research Topic indicates a strong need for further research in the area of PPTMs with particular emphasis on these non-mainstream PPTMs in plant biology such as lysine 2-hydroxyisobutyrylation and succinylation.

## Author contributions

ML: Conceptualization, Data curation, Formal analysis, Project administration, Software, Supervision, Validation, Writing – original draft, Writing – review & editing. SD: Writing – review & editing. ZD: Writing – review & editing. LL: Writing – review & editing.

